# Draft Genome Sequence of a Mycobacterium chelonae subsp. *bovis* Strain Isolated from a Baikal Seal (Pusa sibirica) in Captivity

**DOI:** 10.1128/mra.01135-22

**Published:** 2023-02-22

**Authors:** Takeshi Komine, Yuko Matsuoka, Mari Inohana, Osamu Kurata, Shinpei Wada

**Affiliations:** a Laboratory of Aquatic Medicine, School of Veterinary Medicine, Nippon Veterinary and Life Science University, Tokyo, Japan; b Lake Biwa Museum, Kusatsu, Shiga, Japan; University of Rochester School of Medicine and Dentistry

## Abstract

Mycobacterium chelonae is a nontuberculous mycobacterium that causes infections in various animals, including humans. In this study, we report the draft genome sequence of M. chelonae subsp. *bovis* strain NJB1701, which was isolated from a Baikal seal (Pusa sibirica) in captivity in Japan.

## ANNOUNCEMENT

Mycobacterium chelonae, a ubiquitous, rapidly growing, nontuberculous mycobacterium, causes mycobacteriosis in various animals, including humans (1–7). The genome of M. chelonae strain NJB1701, which was isolated in 2017 from an infected Baikal seal (Pusa sibirica) reared at the Lake Biwa Museum (Kusatsu, Shiga, Japan), was sequenced.

Punch biopsy samples were isolated from the seal’s dorsolumbar ulcerative nodules, which were approximately 17 cm in diameter. The samples were homogenized with 0.5 mL of distilled water and disinfected with 0.75 mL of 1 N HCl for 15 min. They were then neutralized with 0.75 mL of 1 N NaOH and centrifuged at 3,000 × *g* for 20 min. The supernatant was discarded, and the pellet was inoculated in Middlebrook 7H11 agar supplemented with 10% BBL Middlebrook oleic acid-albumin-dextrose-catalase (OADC) enrichment (Becton, Dickinson and Company, USA). The medium was incubated at 25°C for 5 days. The isolate obtained was identified as M. chelonae based on the Runyon classification system (8), salt tolerance, citrate utilization (9, 10), and phylogenetic analysis of three housekeeping genes (11).

A frozen stock solution (stored at −80°C in 20% glycerol) of the isolated strain NJB1701 was streaked on a 2% Ogawa egg slant (Kyokuto Pharmaceutical Industrial Co., Ltd., Japan) and incubated at 25°C for 4 weeks. Subsequently, approximately 1.0 × 10^9^ CFU of NJB1701 was collected, boiled at 95°C for 15 min, frozen at −20°C overnight, and pulverized twice (4,500 rpm for 1 min) with 0.5-mm-diameter zirconia/silica beads (BioSpec Products, Inc., USA) using a MicroSmash MS-100 system (Tomy Digital Biology Co., Ltd., Japan). Genomic DNA was extracted using the NucleoSpin Plant II kit (Macherey-Nagel GmbH & Co. KG, Germany). DNA libraries were prepared using a KAPA HyperPrep kit (Roche, Switzerland) and an MGIEasy universal library conversion kit (App-A) (MGI Tech, China) following the manufacturer’s protocols and were sequenced using a DNBSEQ-G400 sequencer (2 × 150 bp), yielding 17,824,140 reads. The quality of the raw reads was assessed using FastQC v0.11.9 (12). Sequence reads were trimmed using fastp v0.20.1 (13) and assembled *de novo* using Platanus_B v1.1.0 (14). Genome completeness and contamination were assessed using CheckM v1.0.7 (15). Automated annotation was performed using the DNA Data Bank of Japan FAST Annotation and Submission Tool (DFAST) (https://dfast.ddbj.nig.ac.jp). The draft genome was discovered to be 99.48% complete, containing 0.03% contamination, and consisted of 51 contigs, with a total length of 5,162,943 bp, a contig *N*_50_ value of 606,935 bp, a G+C content of 64.1%, and genome coverage of 39×. DFAST identified 5,128 coding sequences, 1 rRNA gene, and 47 tRNA genes.

**FIG 1:**
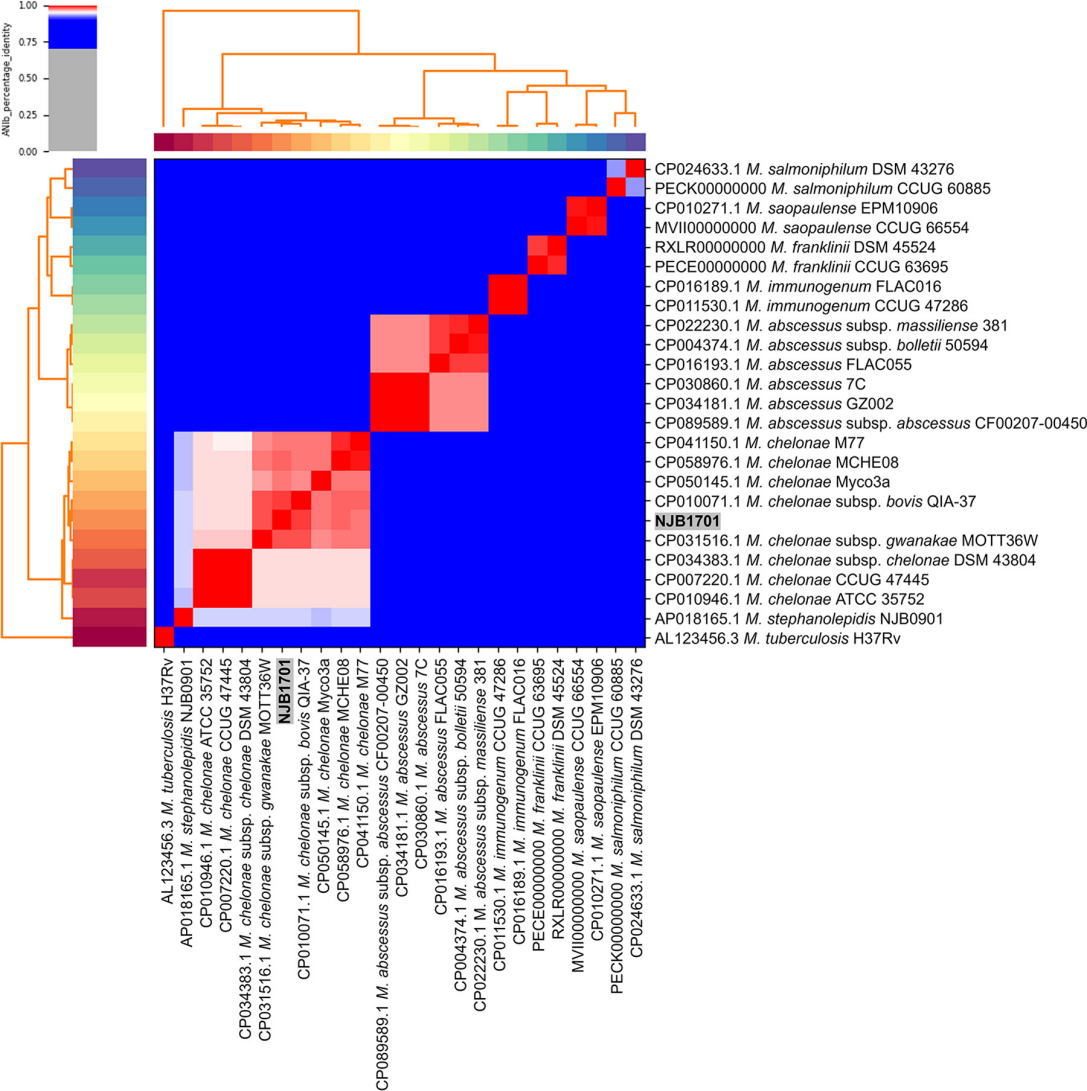
Heatmap of ANI values for 25 Mycobacterium strains. The heatmap was generated using PyANI v0.2.11 with a BLAST-based approach (ANIb). The ANI values for NJB1701 versus other M. chelonae strains were >95.5%. NJB1701 was clustered with M. chelonae subsp. *bovis*, with an ANI value of 98.7%.

Average nucleotide identity (ANI) analysis was conducted using PyANI v0.2.11 (option −m ANIb) (16) to determine the relationships among Mycobacterium species. The ANI analysis revealed that NJB1701 was clustered with M. chelonae subsp. *bovis* (17), with an ANI value of 98.7% (Fig. 1). Default parameters were used for all software unless noted otherwise.

Our findings will improve our understanding of the pathogenicity and evolution of M. chelonae strain NJB1701.

### Data availability.

The raw sequencing data are available in the DDBJ Sequence Read Archive under the accession number DRR409418, the BioSample accession number SAMD00545766, and the BioProject accession number PRJDB14421. The draft genome sequences were submitted to the DDBJ under accession number BSAK01000000.
